# Predicting Youth and Young Adult Treatment Engagement in a Transdiagnostic Remote Intensive Outpatient Program: Latent Profile Analysis

**DOI:** 10.2196/47917

**Published:** 2023-09-07

**Authors:** Kate Gliske, Katie R Berry, Jaime Ballard, Clare Schmidt, Elizabeth Kroll, Jonathan Kohlmeier, Michael Killian, Caroline Fenkel

**Affiliations:** 1 Charlie Health Inc Bozeman, MT United States; 2 Center For Applied Research and Educational Improvement University of Minnesota St. Paul, MN United States; 3 College of Social Work Florida State University Tallahassee, FL United States

**Keywords:** youth, young adult, virtual, mental health, intensive outpatient treatment, latent profile analysis, personalized treatment

## Abstract

**Background:**

The youth mental health crisis in the United States continues to worsen, and research has shown poor mental health treatment engagement. Despite the need for personalized engagement strategies, there is a lack of research involving youth. Due to complex youth developmental milestones, there is a need to better understand clinical presentation and factors associated with treatment engagement to effectively identify and tailor beneficial treatments.

**Objective:**

This quality improvement investigation sought to identify subgroups of clients attending a remote intensive outpatient program (IOP) based on clinical acuity data at intake, to determine the factors associated with engagement outcomes for clients who present in complex developmental periods and with cooccurring conditions. The identification of these subgroups was used to inform programmatic decisions within this remote IOP system.

**Methods:**

Data were collected as part of ongoing quality improvement initiatives at a remote IOP for youth and young adults. Participants included clients (N=2924) discharged between July 2021 and February 2023. A latent profile analysis was conducted using 5 indicators of clinical acuity at treatment entry, and the resulting profiles were assessed for associations with demographic factors and treatment engagement outcomes.

**Results:**

Among the 2924 participants, 4 profiles of clinical acuity were identified: a low-acuity profile (n=943, 32.25%), characterized by minimal anxiety, depression, and self-harm, and 3 high-acuity profiles defined by moderately severe depression and anxiety but differentiated by rates of self-harm (high acuity+low self-harm: n=1452, 49.66%; high acuity+moderate self-harm: n=203, 6.94%; high acuity+high self-harm: n=326, 11.15%). Age, gender, transgender identity, and sexual orientation were significantly associated with profile membership. Clients identified as sexually and gender-marginalized populations were more likely to be classified into high-acuity profiles than into the low-acuity profile (eg, for clients who identified as transgender, high acuity+low self-harm: odds ratio [OR] 2.07, 95% CI 1.35-3.18; *P*<.001; high acuity+moderate self-harm: OR 2.85, 95% CI 1.66-4.90; *P*<.001; high acuity+high self-harm: OR 3.67, 95% CI 2.45-5.51; *P*<.001). Race was unrelated to the profile membership. Profile membership was significantly associated with treatment engagement: youth and young adults in the low-acuity and high-acuity+low–self-harm profiles attended an average of 4 fewer treatment sessions compared with youth in the high-acuity+moderate–self-harm and high-acuity+high–self-harm profiles (*ꭓ*^2^_3_=27.6, *P*<.001). Individuals in the high-acuity+low–self-harm profile completed treatment at a significantly lower rate relative to the other 2 high-acuity profiles (*ꭓ*^2^_3_=13.4, *P*=.004). Finally, those in the high-acuity+high–self-harm profile were significantly less likely to disengage early relative to youth in all other profiles (*ꭓ*^2^_3_=71.12, *P*<.001).

**Conclusions:**

This investigation represents a novel application for identifying subgroups of adolescents and young adults based on clinical acuity data at intake to identify patterns in treatment engagement outcomes. Identifying subgroups that differentially engage in treatment is a critical first step toward targeting engagement strategies for complex populations.

## Introduction

### Background

Youth mental health crises continue to persist and grow across the United States [1,2]. Nearly 20% of youth and young adults have a diagnosed mental health condition [2], whereas 20% have seriously considered suicide in the past year. Marginalized groups such as Indigenous; female; and lesbian, gay, bisexual, transgender, queer (or questioning), asexual (or allied), intersex (LGBTQIA+) youth report even higher rates of suicidality [3].

Psychological interventions can effectively address these mental health concerns, leading to reduced symptoms and improved functioning, productivity, and life span [4,5]. The outcomes are particularly strong with early intervention during adolescence and young adulthood [6,7]. However, many youth and young adults (23%-63%) drop out of treatment before completing the recommended dose [8], leading to a smaller reduction in symptoms and a lower likelihood of maintaining improvement [9]. The purpose of this quality improvement study was to use person-centered analyses to identify client subgroups based on the initial symptom severity of youth and young adult clients of a nationwide telehealth intensive outpatient program (IOP) and to explore differences in treatment engagement between groups.

### Treatment Engagement

Treatment engagement is associated with better treatment outcomes [10,11]. However, mental health treatment dropout rates are high, particularly among younger clients [12]. Across treatment settings and diagnoses, dropout rates generally range between one-fourth and over half of all clients [8]. Although some patients end treatment because they have seen improvement and feel ready to leave early, others drop out because of changes in motivation or perceptions of usefulness [13] or a lack of stability in their lives [14].

Clients who drop out early are less likely to have significant improvements in depression or anxiety [10,11] or to maintain improvement [9] and have smaller reductions in trauma symptoms. Similar patterns were observed in the number of sessions attended; for example, in one study of outpatient depression treatments, each additional session attended was associated with a 20% increased likelihood of clinically significant improvement [11]. Therefore, it is critical that providers identify clients at risk of dropout and implement strategies to increase engagement.

Variable-centered research on individual predictors of dropout among youth and young adults has indicated few reliable pretreatment predictors [15]. Machine learning research on dropout among adults has improved predictive power [12]; however, this research has not been conducted with youth. Patient stratification through group analysis may be ideally positioned to identify youth and young adult patient profiles at a higher risk of dropout and disengagement.

### Patient Stratification and Mental Health Care Engagement

Across health care, extensive efforts are underway to develop methods to predict individual responses to treatments and select the ideal treatment and engagement strategies for the individual, such as decision support algorithms [16]. Within mental health care for adults, these methods are in development, and several have been tested in clinical trials [17,18]. Patient stratification analyses, such as latent class analysis, can identify subgroups of clients at the highest risk of poor mental health treatment engagement [19] or clinical outcomes [20]. Identifying high-risk subgroups can guide the choice of effective treatment strategies [21]; for example, midweek support calls can increase treatment engagement among clients with depression, but this intervention would be costly to implement for all clients [22]. Patient stratification could identify those at the greatest risk for dropout and indicate targeted engagement interventions. This latent class analysis approach allows for the simultaneous prediction of multiple outcomes and may prove to be the optimal method for treatment selection decisions under real-world circumstances [23].

One landmark study used a large data set of adult clients receiving stepped-care treatment for depression and anxiety disorders to identify subgroups and explore whether there were differences in dropout between subgroups, as well as differences in recovery and reliable change [19]. The authors identified and replicated subgroups that robustly distinguished profiles that were most likely to drop out and differences in dropout by class between 2 different interventions. This method of developing profiles has the advantage of seamlessly integrating into current clinical care, where client intake information can be used to identify profile alignment and guide effective treatment and engagement planning.

### Lack of Research on Youth

Despite the potential advantages of tailored treatment and engagement strategies, few studies have specifically assessed the profiles and differential outcomes among youth and young adults [24]. As treatment dropout is higher among youth and young adults, there is a greater need for strategies that help them remain in treatment long enough to benefit [24,25]. Although several engagement strategies have demonstrated effectiveness, researchers have not consistently identified who could benefit the most. A foundational meta-analysis found 3 significant dropout predictors among youth: contact with deviant peers, externalizing problems, and ethnic minority group status [8]. In addition to these factors, research has found few consistent predictors of dropout among youth, although older age and male sex have been reported in some studies [15,25,26].

This challenge in identifying consistent dropout predictors for youth may be partly due to the complex developmental periods spanning early adolescence to young adulthood. Youth experience a barrage of psychosocial and neurobiological changes during this time while simultaneously experiencing increasing environmental and psychosocial pressures [27,28]. The interactions between environmental factors, genetic influences, and the changing brain create a complicated network of influencers that drives the manifestation of mental health disorders [29,30]. Symptoms of mental health disorders often manifest in early adolescence or young adulthood; 74% of people with mental health disorders at the age of 26 years also have a mental health diagnosis before the age of 18 years [29]. Due to the complexity of this population, research suggests the need for developmentally appropriate mental health treatment and engagement strategies that are distinct from that of adults and responsive to unique needs across the stages of development [29]. There is a pressing need to better understand the mechanisms of action that drive successful mental health treatment and engagement of youth and to be able to identify clients at most risk who could benefit from support.

### This Study

This quality improvement study used latent profile analysis (LPA) to identify client subgroups based on the initial symptom severity of youth and young adult clients and sought to identify differences in treatment engagement between profiles. This approach provides a data-driven model for identifying subgroups to personalize treatment and engagement strategies [31].

Drawing from a nationwide remote telehealth IOP, this study provides a practical opportunity to assess subgroups and outcomes in a transdiagnostic IOP among youth and young adults with multiple cooccurring conditions. This addresses a research-practice gap wherein youth mental health providers commonly treat youth with many different diagnoses [32] and at different developmental periods, yet research and best practices for youth mental health treatment have largely focused on homogeneous populations and analyzing single variable predictors [33-35]. The aim of this quality improvement analysis was to (1) identify the clinical acuity profiles of youth and young adults entering web-based intensive outpatient treatment, (2) examine the association between profile membership and demographic characteristics, and (3) test for differences in clinical profiles on measures of treatment engagement.

## Methods

### Setting

The data set used in this investigation was collected as part of routine outcome monitoring undertaken at a national provider of mental health services for youth and young adults. Charlie Health delivers remote-only IOP to individuals aged 11 to 30 years with complex mental health disorders. Client outcomes are routinely collected using validated measures to meet stakeholder and accreditation requirements for ongoing program evaluation to identify opportunities for service quality improvement and to drive increases in treatment responsiveness. This study is part of an organization-wide initiative to identify effective ways to personalize the components of mental health treatment for youth with mental health needs that necessitate IOP treatment.

The client population of Charlie Health typically presents with high clinical acuity, a high prevalence of cooccurring mental and behavioral health issues that impair daily functioning, and significant histories of trauma. Clients often require more intensive services than those that can be delivered in a community-based care setting, or they come from regions lacking mental health resources. Because Charlie Health is in network with both public and private insurers, there is a diverse representation of socioeconomic backgrounds. In addition, clients have widely variable experiences of treatment history before admission to Charlie Health (ie, clients may be stepping down from a higher level of care, stepping up from a lower level of care, or initiating treatment for the first time).

Weekly IOP at Charlie Health involves 9 hours of web-based group sessions paired with optional 1-hour individual or 1-hour family sessions as determined by the needs and willingness of the clients or caregivers to participate. Group sessions are 3 hours long, broken into 50-minute sessions with 10-minute breaks in between. These sessions include experiential therapy (eg, art, music, and journaling); general therapeutic processing; and evidence-based skill-building interventions (eg, dialectical behavioral therapy and cognitive behavioral therapy). Because of the size and reach of Charlie Health and the accessibility of web-based programming, clients can be assigned to group IOP tracks based on shared identities (eg, gender, age, sexual orientation, and race); presenting issues (eg, depression, anxiety, and substance use); and appropriate treatment (eg, trauma-focused cognitive behavioral therapy). To complement the individual, family, and group sessions of the IOP, Charlie Health offers comprehensive family support programs to provide psychoeducational and support groups to caregivers and loved ones. For clients who require psychopharmacological support, a team of psychiatrists and psychiatric mental health nurse practitioners is on staff to conduct evaluations and prescribe medication as needed. In addition, clients are supported by a team of Care Experience Specialists who offer care coordination outside individual or group therapy sessions. Individual and group sessions are offered a variety of times throughout the day to meet the needs of a range of schedules and to offer a higher degree of accessibility. The average length of stay in the program is 10 to 12 weeks. The target length of stay is individually determined by the clinical staff and varies by client acuity and need.

### Participants

The sample for this investigation included Charlie Health clients who were discharged between July 2021 and February 2023 (N=2924) and who completed both an intake and discharge survey. The sample was selected to include multiple demographic factors associated with profile membership in the analyses. Some demographics were asked on the intake survey (race and age), and some were asked on the discharge survey (gender, sexual orientation, and transgender status). The sample included clients who were discharged following the successful completion of treatment as well as those discharged due to disengagement, insurance denial, or referral to a higher or lower level of care.

### Ethics Approval

This program evaluation research was reviewed and approved by the Florida State University Institutional Review Board that deemed this type of investigation “nonhuman subjects research,” given its primary purpose of program evaluation and quality improvement (STUDY00003364).

### Data Collection Procedures

As part of Charlie Health’s routine data collection procedures, data were collected from clients during the first and last IOP sessions. Clients logged into the web-based group room and were then sent to a web-based survey room, where a Charlie Health staff member provided them with a closed survey Qualtrics link to complete either an intake or discharge survey. For any client who missed their last IOP session, the discharge survey link was emailed and texted to them with a small incentive for completion. All client data were downloaded and deidentified before analyses. All clinical symptom measures in this study were collected during intake survey. The measures were presented in the same order for each client.

### Measures

#### Demographics

Demographic data were collected by self-report on both the intake (race and age) and discharge surveys (gender, sexual orientation, and transgender status) and consisted of questions regarding race (*African American or Black, Asian, Indigenous people around the world, Middle Eastern or North African, White,* and *other*); gender (*male, female, nonbinary, genderqueer, nonconforming, gender fluid,* and *gender neutral individuals*); and sexual orientation (*straight, asexual or gray sexuality, bisexual, pansexual, gay, lesbian, queer,* and *questioning*). Clients could multiselect across each question to capture intersectionalities. Clients were also asked whether they identified themselves as transgender (yes or no).

#### Use of High-Acuity Service

To determine the use of high-acuity service before intake, clients were asked to self-report whether they were admitted to the emergency room (ER) or a higher level of care facility (ie, inpatient and residential treatment) in the 30 days before their first group therapy session at Charlie Health. If a client answered yes to either, they were asked for the reason for their admission (*suicidal thoughts, self-harm, suicide attempt, substance use, eating disorder,* and *other*).

#### World Health Organization-5 Well-Being Index

The World Health Organization-5 Well-Being Index (WHO-5) is a self-reported measure of well-being that consists of 5 questions on a scale of 0 (*At no time*) to 5 (*All of the time*). The sum score, ranging from 0 to 25, is multiplied by 4 to give a final score out of 100, with 0 being the worst outcome for well-being and 100 being the best outcome for well-being. A cutoff score of ≤50 is used to assign a screening diagnosis of depression [36].

#### Generalized Anxiety Disorder-7

The Generalized Anxiety Disorder-7 (GAD-7) is a self-reported measure of generalized anxiety symptoms that consists of 7 questions on a scale of 0 (*not at all*) to 3 (*nearly every day*). The total score, ranging from 0 to 21, is calculated. Scores of 5, 10, and 15 represent cutoff points for mild, moderate, and severe anxiety, respectively [37].

#### Patient Health Questionnaire Modified for Adolescents

The Patient Health Questionnaire modified for adolescents (PHQ-A) is a 9-item, self-reported measure of depressive symptoms that consists of questions on a scale of 0 (*not at all*) to 3 (*nearly every day*). The total score, ranging from 0 to 27, is calculated. PHQ-A scores of 5, 10, 15, and 20 represent mild, moderate, moderately severe, and severe depression, respectively [38].

#### Self-Harm

To determine the acuity of self-harm, clients were asked to self-report how many days over the last month before intake that they self-injured and were given a sliding scale from 0 to 30 days.

#### Daily Functioning

To assess how often mental health affected their daily functioning, clients were asked to self-report whether they were enrolled in school or if they were working. If so, they were then asked how many days in the last 7 days their mental health interfered with their ability to attend school or work and were given the option of reporting 0 to 7 days.

#### Treatment Engagement Outcomes

Three treatment engagement outcomes were targeted in the analysis: the total number of groups attended, treatment completion, and disengagement from treatment before completing treatment goals. For the total number of groups attended, each 3-hour block of group therapy was considered one group therapy session. The sum of all group therapy sessions attended was created for each client. Treatment completion was defined as a client completing their treatment goals and being routinely discharged. Disengagement was defined as a client discharging from treatment against clinical recommendations or without completing treatment goals.

### Statistical Methods

#### Overview

We used LPA to uncover homogeneous subgroups of youth and young adults within the larger heterogeneous treatment population who reported similar patterns of responses to a set of observed variables or indicators. In an LPA, means and SDs from continuous indicators are used to estimate the posterior probabilities of belonging to a particular profile [39]. We included a number of variables that were conceptually supported to differentiate clinical acuity in this population. We selected clinical and functional indicator variables that were modeled to arrive at the most parsimonious solution with the best model fit criteria; the model with the 5 indicators is described in subsequent sections.

#### Identify Clinical Acuity Profiles of Youth and Young Adults Entering Virtual IOP

Data preparation was conducted using SPSS Statistics (version 29.0; IBM Corp), and LPA class enumeration and multinomial regressions were conducted in Mplus [40]. The solutions were estimated during the profile enumeration process to determine the optimal number of profiles. Each subsequent profile structure (k) was evaluated and compared with the previous model (k − 1) to determine whether it provided a better relative and absolute fit according to several indicators. These included the log likelihood values; Akaike information criterion [41], Bayesian information criterion (BIC) [42], and a BIC criterion adjusted for sample size, where lower values indicate better model fit; and the Lo-Mendell-Rubin test [43] and Bootstrap Likelihood Ratio test [44], where a failure to reject the null indicates that the current model with k profiles does not substantially improve model fit compared with a prior model with k − 1 profiles. In addition, an entropy score was calculated for each model, where scores closer to 1.0 indicate better profile prediction [45]. Finally, we assessed within-profile homogeneity and between-profile separation using the average posterior class probability, where values >0.70 indicate adequate separation, and the odds of correct classification, where values >5 indicate adequate classification [46].

#### Examine the Association Between Profile Membership and Demographic Characteristics

A modified 3-step multinomial logistic regression analysis (R3STEP multinomial regression) [47,48] was used to assess the demographic and clinical factors associated with latent profile membership. The DU3STEP approach [48] for continuous distal outcomes and the DCAT approach [49] for categorical distal outcomes were used to evaluate the relationship between clinical acuity profiles and various proxies for treatment engagement. The three distal outcome models tested the relationship between membership in each profile at treatment intake and (1) length of stay, (2) treatment completion, and (3) disengagement.

LPA uses full information maximum likelihood with robust SEs by computing the parameter estimates using all available information, which is considered the best practice for data missing at random, as it avoids listwise deletion [50].

#### Treatment Engagement Outcomes Related to Profile Membership

Chi-squared analysis was used to compare profiles and assess the differences in treatment engagement outcomes. Separate analyses were performed for the 3 variables of the total number of sessions attended, treatment completion, and early disengagement.

## Results

### Sample Demographics

The sample included 2924 clients. Participant demographics are displayed in Table 1.

**Table 1:** Demographics of full sample and the 4 classes.

Variable		Full sample (N=2924)	Low-acuity profile (n=943)	High-acuity+low–self-harm profile (n=1452)	High-acuity+moderate–self-harm profile (n=203)	High-acuity+high–self-harm profile (n=326)
Age (years), mean (SD)		17.55 (4.62)	16.99 (4.26)	18.58 (4.99)	16.54 (3.73)	15.51 (3.01)
**Age group (years), n (%)**						
	Youth (<18)	1807 (61.8)	658 (69.8)	738 (50.8)	141 (69.5)	270 (82.8)
	Young adult (18-24)	759 (26)	204 (21.6)	471 (32.4)	47 (23.2)	37 (11.3)
	Adult (≥25)	300 (10.3)	68 (7.2)	215 (14.8)	9 (4.4)	8 (2.5)
	Missing data	58 (2)	13 (1.4)	28 (1.9)	6 (3)	11 (3.4)
**Gender, n (%)**						
	Female	1397 (47.8)	434 (46)	729 (50.2)	93 (45.8)	141 (43.3)
	Male	250 (8.5)	344 (36.5)	301 (20.7)	39 (19.2)	76 (23.3)
	Nonbinary	84 (2.9)	54 (5.7)	129 (8.9)	26 (12.8)	41 (12.6)
	Nonconforming	150 (5.1)	18 (1.9)	45 (3.1)	4 (2)	17 (5.2)
	Gender fluid	61 (2.1)	26 (2.8)	73 (5)	21 (10.3)	30 (9.2)
	Gender questioning	25 (0.9)	11 (1.2)	34 (2.3)	3 (1.5)	13 (4)
	Gender neutral	54 (1.8)	8 (0.8)	12 (0.8)	1 (0.5)	4 (1.2)
	Multiple	33 (1.1)	9 (1)	40 (2.8)	5 (2.5)	0 (0)
	Other	110 (3.8)	10 (1.1)	18 (1.2)	4 (2)	1 (0.3)
	Missing data	250 (8.5)	29 (3.1)	71 (4.9)	7 (3.4)	3 (0.9)
**Race, n (%)**						
	Asian	38 (1.3)	9 (1)	22 (1.5)	5 (2)	3 (0.9)
	Black	119 (4.1)	45 (4.8)	62 (4.3)	8 (3.9)	4 (1.2)
	Indigenous people around the world	26 (0.9)	9 (1)	9 (0.6)	5 (2.5)	3 (0.9)
	Middle Eastern or North African	6 (0.2)	1 (0.1)	3 (0.2)	0 (0)	2 (0.6)
	White	874 (29.9)	277 (29.4)	525 (36.2)	55 (27.1)	17 (5.2)
	Mixed race	94 (3.2)	26 (2.8)	59 (4.1)	8 (3.9)	1 (0.3)
	Other	79 (2.7)	31 (3.3)	44 (3)	2 (1)	2 (0.6)
	Missing data	1688 (57.5)	545 (57.8)	728 (50.1)	121 (59.6)	294 (90.2)
**Ethnicity, n (%)**						
	Hispanic or Latino	247 (8.4)	85 (9)	137 (9.4)	15 (7.4)	10 (3.1)
	Not Hispanic or Latino	761 (26)	234 (24.8)	462 (31.8)	48 (23.6)	17 (5.2)
	Other	66 (2.3)	30 (3.2)	30 (2.1)	4 (2)	2 (0.6)
	Missing data	1850 (63.3)	594 (63)	823 (56.7)	136 (67)	297 (91.1)
**Sexual orientation, n (%)**						
	Asexual or gray sexual	118 (4)	27 (2.9)	56 (3.9)	12 (5.9)	23 (7.1)
	Bisexual	621 (21.2)	173 (18.3)	322 (22.2)	50 (24.6)	76 (23.3)
	Pansexual	38 (13.1)	82 (8.7)	191 (13.2)	33 (16.3)	76 (23.3)
	Gay	79 (2.7)	22 (2.3)	35 (2.4)	6 (3)	16 (4.9)
	Heterosexual or straight	992 (33.9)	430 (45.6)	452 (31.1)	49 (24.2)	61 (18.7)
	Lesbian	138 (4.7)	41 (4.3)	74 (5.1)	9 (4.4)	14 (4.3)
	Queer	137 (4.7)	23 (2.4)	73 (5)	13 (6.4)	28 (8.6)
	Questioning	135 (4.6)	40 (4.2)	66 (4.5)	8 (3.9)	21 (6.4)
	Other	158 (5.4)	47 (5)	91 (6.2)	14 (6.9)	6 (1.8)
	Missing data	164 (5.6)	58 (6.2)	92 (6.3)	9 (4.4)	5 (1.5)
**Recent mental health treatment history, n (%)**						
	Higher level of care use 30 days before admission	883 (30.2)	319 (33.8)	349 (24)	78 (38.4)	137 (42)
	ER^a^ use 30 days before admission	835 (28.6)	226 (24)	388 (26.7)	77 (37.9)	144 (44.2)
**Indicators, mean (SD)**						
	Depressive symptoms	14.32 (7.22)	6.56 (4.20)	17.95 (4.67)	18.21 (6.26)	18.47 (5.93)
	Anxiety symptoms	12.14 (6.13)	5.30 (3.34)	15.57 (3.79)	15.21 (5.21)	15.43 (4.67)
	Days of self-harm	5.80 (10.12)	0.52 (1.68)	1.12 (1.90)	13.89 (3.69)	29.18 (1.89)
	Symptom interference with daily functioning	2.78 (2.59)	1.92 (2.37)	3.06 (2.62)	3.49 (2.45)	5.00 (2.18)
	Psychological well-being	32.99 (22.72)	55.69 (21.81)	22.57 (13.15)	20.43 (14.58)	20.80 (14.58)
**Engagement outcomes**						
	Total number of IOP^b^ group sessions attended, mean (SD)	25.06 (13.22)	23.98 (12.91)	24.82 (13.36)	27.44 (13.93)	27.83 (12.50)
	IOP treatment completion, n (%)	1804 (61.7)	580 (61.5)	876 (60.3)	134 (66)	214 (65.6)
	Early disengagement from IOP, n (%)	644 (22)	228 (24.2)	344 (23.7)	40 (19.7)	32 (9.8)

^a^ER: emergency room.

^b^IOP: intensive outpatient program.

### Aim 1: Identify Clinical Acuity Profiles of Youth and Young Adults Entering Web-Based IOP

**Figure 1:**
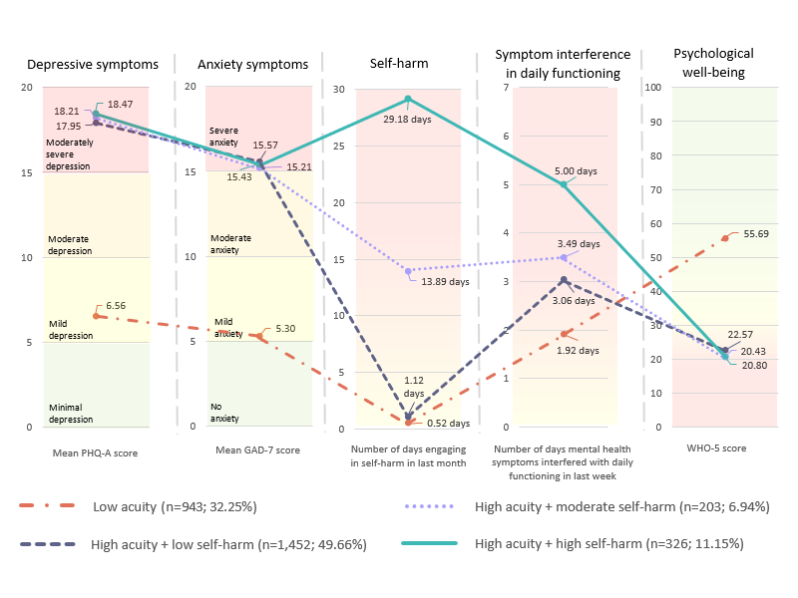
The 4-profile solution of baseline clinical acuity (N=2924). GAD-7: Generalized Anxiety Disorder-7; PHQ-A: Patient Health Questionnaire modified for adolescents; WHO-5: World Health Organization-5 Well-Being Index.

Tables 2 and 3 display the fit statistics and classification diagnostics from the class enumeration process for 1 to 5 latent profile solutions. As shown in Table 2, there were diminishing returns in the Akaike information criterion, BIC, and sample-size adjusted BIC fit statistics. The nonsignificant Lo-Mendell-Rubin value for the 5-profile solution (*P*=.06) indicates that the 4-profile model should be accepted. The 4-profile model was also the most conceptually parsimonious, as the 5-profile solution displayed an artificial division of 1 profile into 2 new profiles. These results collectively indicate a 4-profile solution as the best fit (Figure 1). The profile interpretation is outlined in the text below.

**Table 2:** Results of the latent class enumeration and measures of absolute and relative fit of latent classes among youth and young adults in remote intensive outpatient program (N=2924).

Profiles	Log likelihood	AIC^a^	BIC^b^	Sample-size adjusted BIC	Entropy	LMR^c^, *P* value	BLRT^d^, *P* value
1	−28,816.765	57,653.530	57,713.337	57,681.563	N/A^e^	N/A	N/A
2	−27,202.150	54,436.301	54,531.992	54,481.154	0.888	<.001	<.001
3	−26,290.085	52,624.170	52,755.746	52,685.844	0.812	<.001	<.001
4	−25,483.181	51,022.363	51,189.823	51,100.856	0.831	<.001	<.001
5	−25,197.586	50,463.171	50,666.515	50,558.485	0.834	.06	<.001

^a^AIC: Akaike information criterion.

^b^BIC: Bayesian information criterion.

^c^LMR: Lo-Mendell-Rubin.

^d^BLRT: Bootstrap Likelihood Ratio test.

^e^N/A: not applicable.

**Table 3:** Model classification diagnostics of the 4-class solution (relative entropy, Ek=0.84) among youth and young adults in remote intensive outpatient program (N=2924).

Class k	p_k_^a^	mcaP_k_^b^	AvePP_k_^c^	OCC_k_^d^
Class 1	0.32914	0.32914	0.923	24.43
Class 2	0.12662	0.12662	0.999	6890.75
Class 3	0.46605	0.46605	0.878	8.25
Class 4	0.0782	0.0782	0.959	275.72

^a^p_k_: model estimated proportion for class k.

^b^mcaP_k_: modal class assignment proportion for class k.

^c^AvePP_k_: average posterior probability for class k.

^d^OCC_k:_ odds of correct classification.

#### Low Acuity

Profile 1 (943/2924, 32.25%) consisted of individuals who were admitted to treatment reporting mild levels of depression (PHQ-A: mean 6.56, SD 4.20) and anxiety (GAD-7: mean 5.30, SD 3.34) and minimal self-harm (0.52, SD 1.68 days of last 30 days). Youth and young adults in this profile reported lesser, yet still meaningful, interference from their mental health symptoms in their daily functioning (1.92, SD 2.37 out of 7 days in the last week). They also endorsed psychological well-being (WHO-5: mean 55.69, SD 21.81) above the cutoff used to detect a screening diagnosis of depression (ie, ≤50) [38].

#### High Acuity+Low Self-Harm

Profile 2 (1452/2924, 49.66%) included individuals who endorsed significantly higher levels of depression and anxiety and lower levels of psychological well-being (WHO-5: mean 22.57, SD 13.15) at treatment entry relative to the low-acuity profile. These youth reported moderately severe depression (PHQ-A: mean 17.95, SD 4.67) and severe anxiety (GAD-7: mean 15.57, SD 3.79). In contrast to their mental health symptom severity, these youth endorsed minimal self-harm (1.12, SD 1.90 days on average out of the last 30 days) in the last month and reported that their mental health symptoms interfered with their daily functioning for 3.06 (SD 2.62) days on average out of the last 7 days.

#### High Acuity+Moderate Self-Harm

Profile 3 (203/2924, 6.94%) reported similar levels of depression and anxiety to profiles 2 and 4, with youth who entered the treatment reporting moderately severe depression (PHQ-A: mean 18.21, SD 6.26) and severe anxiety (GAD-7: mean 15.21, SD 5.21). In contrast to profiles 1 and 2, these youth endorsed self-harm about half of the days in the last month (13.89, SD 3.69 days on average of the last 30 days) and reported that their mental health symptoms interfered with their daily functioning about half the days in the last week (3.49, SD 20.43 days on average of the last 7 days). Finally, similar to profile 2, they reported low levels of psychological well-being (WHO-5: mean 20.43, SD 14.58).

#### High Acuity+High Self-Harm

Profile 4 (326/2924, 11.15%) showed the most consistently poor clinical and functional outcomes across all the profiles. Similar to the individuals in profiles 2 and 3, they entered treatment reporting moderately severe depression (PHQ-A: mean 18.47, SD 5.93) and severe anxiety (GAD-7: mean 15.43, SD 4.67). These youth reported engaging in self-harm nearly every day in the last month (29.18, SD 1.89 days on average) and reported the highest levels of interference with their daily functioning (5, SD 2.18 days on average out of the last 7 days). Correspondingly, they reported the lowest levels of psychological well-being (WHO-5: mean 20.80, SD 14.58).

### Aim 2: Examine the Association Between Profile Membership and Demographic Characteristics

#### Overview

Next, we used multinomial logistic regression to assess various factors associated with latent profile membership, including both demographic history and mental health treatment characteristics, to better understand the structure of each profile (see Table 4 for model parameters of fully adjusted results, using the low-acuity profile as the reference group).

**Table 4:** Odds of latent class membership across demographic and mental health treatment history characteristics at intensive outpatient program admission.

			Low-acuity profile vs high-acuity+low–self-harm profile					Low-acuity profile vs high-acuity+moderate–self-harm profile					Low-acuity profile vs high-acuity+high–self-harm profile		
			B (SE)		OR^a^ (95% CI)		B (SE)			OR (95% CI)		B (SE)			OR (95% CI)
Higher level of care use in last 30 days			−0.68^b^ (0.12)		0.51 (0.40-0.64)		0.42^b^ (0.18)			1.53 (1.08-2.16)		0.57^b^ (0.15)			1.77 (1.34-2.35)
ER^c^ use in last 30 days			0.14 (0.12)		1.15 (0.91-1.45)		0.73^b^ (0.17)			2.06 (1.48-2.88)		0.97^b^ (0.14)			2.63 (2.00-3.46)
Age			0.10^b^ (0.01)		1.11 (1.08-1.13)		−0.02 0.02 (0.02)			0.99 (0.95-1.02)		−0.10^b^ (0.02)			0.91 (0.87-0.94)
**Gender**															
	Female	0.25 (0.15)		1.29 (0.96-1.71)		−0.11 (0.21)			0.90 (0.59-1.37)		−0.29 (0.15)			0.75 (0.56-1.01)	
	Male	−0.79^b^ (0.17)		0.46 (0.33-0.63)		−0.78^d^ (0.25)			0.46 (0.28-0.75)		−0.72^b^ (0.17)			0.49 (0.35-0.68)	
	Nonbinary	0.73^b^ (0.20)		2.08 (1.39-3.10)		1.19^b^ (0.26)			3.28 (1.99-5.41)		1.34^b^ (0.19)			3.82 (2.62-5.58)	
	Transgender	0.73^d^ (0.22)		2.07 (1.35-3.18)		1.05^b^ (0.28)			2.85 (1.66-4.90)		1.30^b^ (0.21)			3.67 (2.45-5.51)	
**Sexual orientation**															
	Bisexual	0.15 (0.17)		1.16 (0.83-1.62)		0.53^e^ (0.24)			1.70 (1.07-2.69)		0.98^b^ (0.18)			2.68 (1.88-3.82)	
	Pansexual	0.26 (0.23)		1.30 (0.83-2.03)		0.66^e^ (0.30)			1.93 (1.07-3.48)		1.70^b^ (0.21)			5.48 (3.61-8.34)	
	Heterosexual	−0.76^b^ (0.13)		0.47 (0.36-0.60)		−0.74^d^ (0.21)			0.48 (0.31-0.73)		−0.65^b^ (0.17)			0.52 (0.38-0.72)	
**Race**															
	Asian	−0.03 (0.12)		0.97 (0.77-1.24)		0.13 (0.17)			1.13 (0.73-2.01)		0.32 (0.28)			1.37 (0.79-2.39)	
	Black	−0.08 (0.37)		0.92 (0.45-1.88)		0.38 (0.52)			1.46 (0.53-4.04)		0.94 (0.85)			2.57 (0.49-13.59)	
	Indigenous people around the world	−0.04 (0.18)		0.96 (0.67-1.37)		0.19 (0.26)			1.21 (0.73-2.01)		0.47 (0.43)			1.60 (0.70-3.87)	
	Middle Eastern or North African	−0.02 (0.10)		0.98 (0.82-1.17)		0.09 (0.13)			1.10 (0.85-1.42)		0.24 (0.21)			1.27 (0.84-1.92)	
	White	−0.01 (0.06)		0.99 (0.88-1.11)		0.06 (0.09)			1.07 (0.90-1.26)		0.16 (0.14)			1.17 (0.89-1.55)	

^a^OR: odds ratio.

^b^*P*<.001.

^c^ER: emergency room.

^d^*P*<.01.

^e^*P*<.05.

#### ER Visit 30 Days Before IOP Admission

Relative to clients in the low-acuity profile, individuals in the high-acuity+moderate–self-harm (odds ratio [OR] 2.06, 95% CI 1.48-2.88; *P*<.001) and high-acuity+high–self-harm (OR 2.63, 95% CI 2.00-3.46; *P*<.001) profiles had significantly higher odds of visiting an ER in the 30 days before their admission, whereas there was no significant difference in ER visit between the high-acuity+low–self-harm profile and the low-acuity profile (OR 1.15, 95% CI 0.91-1.45; *P*=.25).

#### Higher Level of Care Stay Within 30 Days of IOP Admission

Clients in the high-acuity+low–self-harm profile had significantly lower odds of reporting a higher level of care stay in the 30 days before admission than the clients in the low-acuity profile (OR 0.51, 95% CI 0.40-0.64; *P*<.001), whereas clients in the high-acuity+moderate–self-harm (OR 1.53, 95% CI 1.08-2.16; *P*<.001) and high-acuity+high–self-harm (OR 1.77, 95% CI 1.34-2.35; *P*<.001) profiles had significantly higher odds of reporting a higher level of care admission in the month preceding admission to IOP.

#### Age

Age was significantly related to the likelihood of profile memberships, with increased age of the individual associated with a lower likelihood of membership in the high-acuity+high–self-harm group compared with the low-acuity profile group (OR 0.91, 95% CI 0.87-0.94; *P*<.001). Increased age was significantly related to classification in the high-acuity+low–self-harm profile relative to the low-acuity profile (OR 1.11, 95% CI 1.08-1.13; *P*<.001). Age was not significantly associated with membership in the high-acuity+moderate–self-harm profile relative to the low-acuity profile (OR 0.99, 95% CI, 0.95-1.02; *P*=.45).

#### Gender

In terms of gender, identifying as a female individual was not related to profile membership. A significantly smaller proportion of male individuals were classified in all high-acuity profiles relative to the low-acuity profile (high acuity+low self-harm: OR 0.46, 95% CI 0.33-0.63; *P*<.001; high acuity+moderate self-harm: OR 0.46, 95% CI 0.28-0.75; *P*=.002; high acuity+high self-harm: OR 0.49, 95% CI 0.35-0.68; *P*<.001). Alternatively, a significantly larger proportion of clients who were identified as nonbinary were classified as having high-acuity profiles relative to those with low-acuity profiles (high acuity+low self-harm: OR 2.08, 95% CI 1.39-3.10; *P*<.001; high acuity+moderate self-harm: OR 3.28, 95% CI 1.99-5.41; *P*<.001; high acuity+high self-harm: OR 3.82, 95% CI 2.62-5.58; *P*<.001).

#### Transgender Status

Transgender identification was significantly associated with profile membership in all the high-acuity profiles relative to the low-acuity profile (high acuity+low self-harm: OR 2.07, 95% CI 1.35-3.18; *P*<.001; high acuity+moderate self-harm: OR 2.85, 95% CI 1.66-4.90; *P*<.001; high acuity+high self-harm: OR 3.67, 95% CI 2.45-5.51; *P*<.001).

#### Sexual Orientation

Clients who identified as heterosexual were less likely to be classified in the high-acuity profiles than in the low-acuity profile (high acuity+low self-harm: OR 0.47, 95% CI 0.36-0.60; *P*<.001; high acuity+moderate self-harm: OR 0.48, 95% CI 0.31-0.73; *P*=.001; high acuity+high self-harm: OR 0.52, 95% CI 0.38-0.72; *P*<.001). Identification as bisexual or pansexual was not related to profile membership in the high-acuity+low–self-harm profile relative to the low-acuity profile. However, a significantly greater proportion of clients who identified as bisexual or pansexual were classified into the high-acuity+moderate–self-harm (bisexual: OR 1.70, 95% CI 1.07-2.69; *P*=.02; pansexual: OR 1.93, 95% CI 1.07-3.48; *P*=.03) and high-acuity+high–self-harm profiles (bisexual: OR 2.68, 95% CI 1.88-3.82; *P*<.001; pansexual: OR 5.48, 95% CI 3.61-8.34; *P*<.001).

#### Race

Race was unrelated to profile membership across the 5 racial categories and 4 profiles.

### Aim 3: Treatment Engagement Outcomes Related to Profile Membership

#### Total Number of Group Sessions Attended

Youth and young adults in the low-acuity (23.98 sessions) and high-acuity+low–self-harm (24.82 sessions) profiles attended significantly fewer treatment sessions on average relative to youth in the high-acuity+moderate–self-harm (27.44 sessions) and high-acuity+high–self-harm (27.83 sessions) profiles (*ꭓ*^2^_3_=27.6, *P*<.001)

#### Treatment Completion

Youth and young adults in the high-acuity+low–self-harm profile (876/1452, 60.33%) completed treatment at a significantly lower rate compared with those in the high-acuity+moderate–self-harm profile (134/203, 66%) and those in the high-acuity+high–self-harm profile (214/326, 65.6%; *ꭓ*^2^_3_=13.4, *P*=.004). Individuals in the low-acuity profile (580/943, 61.5%) did not differ significantly in their rates of treatment completion from any of the high-acuity profiles.

#### Disengagement From Treatment Before Completing Treatment Goals

Youth and young adults in the high-acuity+high–self-harm profile (32/326, 9.8%) were significantly less likely to disengage early from treatment than youth within the low-acuity (228/943, 24.2%), high-acuity+low–self-harm (344/1452, 23.69%), and the high-acuity+moderate–self-harm (40/203, 19.7%) profiles (*ꭓ*^2^_3_=71.1, *P*<.001).

## Discussion

### Principal Findings

This quality improvement investigation sought to use person-centered subgroup analyses to identify hidden clusters of youth and young adult clients admitted to a remote IOP who were differentially engaged in treatment based on shared clinical severity indicators. Four profiles were identified, with separation most evident in reported depression and anxiety symptoms (low acuity vs high acuity) and in the number of days spent engaging in self-harm the month before intake (low, moderate, or high).

The low-acuity profile was notable for relatively milder depressive, anxiety, and self-harm symptoms. This reflects similarities to the low-acuity class in stratified analysis with adults, with low depression and anxiety, a long duration, and a low likelihood of chronic symptom outcomes [21]. Although this might indicate a group ill-suited to the considerable time demands of IOP, this profile was twice as likely to report having a higher level of care stay in the month before admission to IOP relative to the high-acuity+low–self-harm profile. It is possible that these clients were previously stabilized in a residential treatment setting and used a remote IOP for their step-down level of care to prevent readmission. As a result, these clients may have required fewer sessions to reach their treatment goals. This is consistent with the finding that youth in this profile attended the fewest sessions but had no substantial differences in treatment completion relative to the other profiles.

Although previous analyses have identified subgroups based on the levels of mental health acuity at intake [19,21], this is the first study to our knowledge to include self-harm. Self-harm is a distinguishing feature among classes; for example, the low-acuity profile and high-acuity+low–self-harm profile were not significantly different for any outcome. Although these 2 profiles reported significantly different levels of depression and anxiety at intake, they both reported very little self-harm (0.52 and 1.12 days on average in the last month). This could suggest that these profiles are more similar than they originally appeared based on their PHQ-A and GAD-7 scores alone, perhaps indicating that the presence of maladaptive coping techniques (such as self-harm) serves as a better clinical indicator of acuity to personalize support in programming.

One of the key variables that differentiated clinical acuity was the number of days in the month before intake that the client self-harmed. It was unexpected that the profile representing clients who reported daily self-harm tied for the highest rates of treatment completion (66%) and had the lowest levels of early disengagement (9.8%) compared with any of the other profiles analyzed. High-acuity clients are typically the most challenging to engage and retain in treatment programs. The tailored curriculum approach used in Charlie Health considers these factors and proactively assigns clients to a type of treatment that is expected to work best for their needs. This finding suggests that the clients classified into the high-acuity+high–self-harm profile maintain high engagement with the program.

Second, in line with previous research [51-53], clients identified as LGBTQIA+ were significantly more likely to be classified into one of the high-acuity profiles relative to the low-acuity profile. Specifically, clients who were identified as nonbinary (OR 3.82, 95% CI 2.62-5.58), transgender (OR 3.67, 95% CI 2.45-5.51), bisexual (OR 2.68, 95% CI 1.88-3.82), and pansexual (OR 5.48, 95% CI 3.61-8.34) had the highest odds of being classified as having a high-acuity+high–self-harm profile, indicating significantly higher acuity for these populations at intake relative to their cis-gender, heterosexual peers. Programs that fail to consider the unique needs of LGBTQIA+ clients may overlook important factors that correlate with symptom severity. In contrast to previous research, ethnic minority group status, gender, and age were not more likely to influence the classification into high-acuity profiles 3 or profiles with higher dropout rates [15,26,54].

### Practice Implications

Treatment providers have been working for decades to unlock the key to personalizing treatment engagement strategies within large care systems. Attention to treatment engagement and those who drop out of treatment is also critical for effective research and evaluation, as clients who drop out often have distinct feature outcomes from those who remain [55]. This analysis was part of an organization-wide initiative to better understand what types of youth and young adult clients enter an IOP level of care and what risk factors for dropout and disengagement may be identified in this setting. Instead of subjectively determining these criteria based on anecdotal evidence alone, using identifying markers that are universally screened has allowed this treatment provider to begin to target specific supports and curriculum adjustments based on a constellation of attributes.

In practice, many treatment providers find it difficult to personalize treatment and engagement strategies according to the unique needs of each client, even though each distinct program is likely not equally efficacious for all. This process is further complicated when working with youth, as there is increased heterogeneity due to the varying levels of development in each age group. The value of this type of investigation lies not in the replication of these particular profiles, which may or may not translate to other populations or treatment settings, but in the systematic approach to identifying subgroups of clients based on key clinical indicators that can be applied anywhere. This inquiry details one such way to harness the outcome variables that treatment providers are already collecting into actionable targets for personalization that are unique to a specific setting and treatment population.

Within this care setting and population, the findings suggest immediate implications. The high-acuity+low–self-harm group is less likely to complete treatment, suggesting that youth with this profile are candidates for additional engagement strategies. The high-acuity+high–self-harm group is less likely to disengage early, suggesting that current engagement strategies for this group are effective, and greater attention on engagement strategies can be focused on the other groups. Furthermore, the finding that the low-acuity profile attended fewer sessions but had no differences in treatment completion based on treatment goals suggests that they need fewer sessions to achieve their treatment goals. This finding can guide general expectations regarding the length of stay for clients with different acuity levels.

### Limitations

Several considerations should be considered when determining the generalizability of the findings of this investigation. Given the heterogeneity of youth and young adult treatment populations, the 4 distinct profiles of clinical acuity at intake may not replicate fully across other treatment providers. These analyses highlight one set of indicators that could be used to understand acuity at intake, but they are by no means exhaustive. Other mental health care providers should endeavor to use their own client data in similar person-centered analyses to uncover solutions that are personalized to their given treatment population. Second, although this evaluation details the identification of discrete profiles, it does not address the application of these profiles to adjust treatment journeys accordingly. Future investigations should build on these findings by helping fine-tune the appropriate levels of support and deployment of further personalized curricula. Even within a well-defined level of care such as IOP, there exists a gradient of ancillary support services. This provides an opportunity to assess the dosage of care and its impact more precisely across different subgroups of patients entering treatment with different clinical and relational needs. Clinical outcome data were not yet available, so this study could not assess the profile association with clinical outcomes; such analysis in the future could guide treatment strategies as well as engagement strategies.

In addition to these broad areas for future research, this study has several methodological limitations. All data are self-reported and so may be subject to client bias. There are limited demographic variables available, and potentially key variables for engagement, such as life stress, are not captured. In addition, the indicators have different recall periods, leading to measurement inconsistencies.

### Conclusions

This quality improvement study identified subgroups of adolescents and young adults based on clinical acuity data at intake to identify trends in treatment engagement outcomes. Identifying barriers and protective factors against disengagement is crucial in providers’ ongoing efforts to ensure that an adequate dose of mental health treatment is delivered. The profiles identified in this study showcase further personalization of treatment and engagement strategies across a complex population and represent a novel application of distilling salient tailoring variables around which to build additional resources and support.

## Abbreviations

BIC: Bayesian information criterion
ER: emergency room
GAD-7: Generalized Anxiety Disorder-7
IOP: intensive outpatient program
LGBTQIA+: lesbian, gay, bisexual, transgender, queer (or questioning), asexual (or allied), intersex
LPA: latent profile analysis
OR: odds ratio
PHQ-A: Patient Health Questionnaire modified for adolescents
WHO-5: World Health Organization-5 Well-Being Index

## Data Availability

Owing to the quality improvement nature of this study, participants did not agree to their data being publicly shared; therefore, supporting data are not available.
